# Characterizing Telehealth Barriers and Preferences to Promote Acceptable Implementation Strategies in Central Uganda: Multilevel Formative Evaluation

**DOI:** 10.2196/60843

**Published:** 2025-01-23

**Authors:** Michael Kizito, Erina Nabunjo Mugabi, Sabrina Ford, Bree Holtz, Kelly Hirko

**Affiliations:** 1 Department of Computer Science, Makerere University Kampala, Kampala, Uganda; 2 Department of Health Policy Planning and Management, School of Public Health, Makerere University Kampala, Kampala, Uganda; 3 College of Human Medicine, Michigan State University, East Lansing, MI, United States; 4 Department of Advertising & PR, College of Communication Arts & Sciences, Michigan State University, East Lansing, MI, United States; 5 Department of Epidemiology and Biostatistics, College of Human Medicine, Michigan State University, East Lansing, MI, United States

**Keywords:** telehealth, telemedicine, health care, disparities, technology, barriers, resource-limited, preferences, Uganda, Africa, barrier, formative evaluation, health service provider, primary care, satisfaction, Sub-Saharan Africa, survey, utility

## Abstract

**Background:**

Telehealth approaches can address health care access barriers and improve care delivery in resource-limited settings around the globe. Yet, telehealth adoption in Africa has been limited, due in part to an insufficient understanding of effective strategies for implementation.

**Objective:**

This study aimed to conduct a multi-level formative evaluation identifying barriers and facilitators for implementing telehealth among health service providers and patients in Central Uganda.

**Methods:**

We collected surveys characterizing telehealth perceptions, barriers, and preferences from health care providers and patients seeking primary care in the Central Region of Uganda from January 2022 to July 2022. Survey development was informed by the technology acceptance model and evaluated predictors of technology acceptance (ie, perceived usefulness, ease of use, and attitudes). We used descriptive statistics to characterize telehealth perceptions and examined differences according to provider and patient characteristics using Student *t* tests.

**Results:**

Nearly 79% (n=48) of 61 providers surveyed had used telehealth, and perceptions were generally favorable. While 93.4% (n=57) reported that telehealth adds value to clinical practice, less than half (n=30, 49.2%) felt telehealth was more efficient than in-person visits. Provider-reported barriers to telehealth included technology challenges for the patient (34/132, 26%), low patient engagement (25/132, 19%), and lack of implementation support (24/132, 18%). Telehealth use was lower among the 91 surveyed patients, with only 19.8% (n=18) having used telehealth. Although 89% (n=81) of patients reported saving time with telehealth approaches, 33.3% (n=30) of patients reported that telehealth made them feel uncomfortable, and 43.8% (n=39) reported concerns about confidentiality. Over 72% (n=66) of patients who had used telehealth previously reported satisfaction with the telehealth services they received. Several differences in perceptions of telehealth according to patient’s self-reported health status were observed.

**Conclusions:**

Perceptions of telehealth were generally favorable, although higher among providers than patients. Barriers impeding telehealth use include technology challenges and the lack of infrastructure and implementation support. Findings from this study can inform the implementation of acceptable telehealth approaches to address disparities propagated by health care access barriers in Sub-Saharan Africa.

## Introduction

Access to quality health care services is critical to achieving optimal health outcomes and reducing health disparities [1]. Individuals residing in resource-limited settings often encounter various economic, social, cultural, and geographic barriers to health care, including provider shortages, inadequate health insurance coverage, transportation challenges, and limited health literacy [2-4]. Health care access barriers are especially stark in many countries in Sub-Saharan Africa, which have 0.23 doctors for every 10,000 people, compared to the highest ratio of 84.2 doctors in the most high-income countries [5]. Increasing access to quality health care and preventive services via telehealth is a promising strategy to reduce health disparities in low- and middle-income countries.

Telehealth refers broadly to the remote delivery of health care services using electronic and telecommunications technology, including voice, chat, and video-based platforms. There are numerous types of telehealth applications, such as live video conferencing for the provision of clinical services to patients (ie, telemedicine), provider-to-provider health care consultation, and remote delivery of nonclinical services, including the use of mobile health (mHealth) tools to support health promotion and wellbeing efforts [6]. Telehealth approaches offer a practical and often cost-effective method to expand the reach of health care services and address health care access barriers in resource-limited settings [7]. Despite the widespread adoption of mobile phones and other digital tools that can support telehealth applications, and the acknowledged potential of using these technologies to improve health care service delivery [8], telehealth adoption in clinical settings remains low. This is particularly true in health care professional shortage areas, which arguably stand to benefit most from these technologies. Indeed, results from a recent review demonstrate the promise of telemedicine approaches in revolutionizing health care delivery in Africa by improving access, efficiency, and patient outcomes [9]. Yet, significant challenges related to the effective implementation and adoption of telehealth remain.

The COVID-19 pandemic highlighted the need for innovative, cost-effective, technology-enabled systems for health care delivery and resulted in a rapid increase in telehealth adoption worldwide. Indeed, the rise in innovative telehealth start-up companies is beginning to transform health care access across Africa, despite concerns about data privacy, inadequate infrastructure, and a lack of policy frameworks [9,10]. For example, Rocket Health based in Kampala, Uganda [11], offers telehealth services as a licensed and registered clinic, laboratory, and pharmacy, including doctor consultations through phone calls and SMS text messages, as well as laboratory sample pickups and medicine deliveries. However, these services can be costly and may not be accessible to those residing in rural areas of Africa with limited internet access. Thus, continued efforts are urgently needed to ensure the broad and equitable reach of telehealth applications in Africa.

An insufficient understanding of effective strategies for implementation has limited telehealth adoption in under-resourced settings. Indeed, very little is known about specific challenges and preferences for telehealth program implementation in African countries, particularly in Uganda [12]. This dearth of knowledge severely limits the ability to implement effective and appropriate telehealth solutions to improve health care access in Africa equitably. Thus, the purpose of this study was to conduct a multi-level formative evaluation identifying barriers and facilitators for implementing telehealth among health service providers and patients in Central Uganda.

## Methods

### Study Population

The study population included health service providers (ie, doctors, nurses, midwives, and lab technicians) and patients seeking health care services at regularly scheduled clinic visits between January and July 2022 at the following health care facilities in the Central Region of Uganda: Namulonge Health Centre III, Wattuba Health Centre III, Kasangati Health Centre IV, and Buwambo Health Centre IV. Uganda’s health care system works on a referral basis and includes small village health teams, health centers (II, III, and IV), district general hospitals, and regional and national referral hospitals, with more intensive services provided at larger centers or hospitals [13]. A health center III facility has approximately 18 staff, including a senior clinical officer, and provides laboratory services and a general outpatient clinic and maternity ward. Health centers IV are small hospitals, with all of the services available at health center III, along with additional medical providers, the ability to admit patients, and a theater for surgical procedures. Although phone and internet access is widespread in Uganda’s health center IV facilities, telemedicine integration is minimal, and technology has been used largely in referral hospital settings to initiate referrals and consultations and for knowledge sharing between providers [14]. Of note, the adoption of telecommunication technologies in Uganda is relatively low, with mobile phone coverage at approximately 60% and mobile internet subscriptions at around 9% in 2016, and primarily among urban residents [15]. Although marked variations in internet coverage across different regions in Uganda are evident, the vast majority of the population in Central Uganda has access to at least 2G networks [16].

### Study Design

We used a cross-sectional study design engaging providers and patients through surveys using a convenience sampling approach. Inclusion criteria required participants to be providers at the study sites or patients receiving health care at the study sites at the time of study recruitment. Study participants were not selected based on prior telehealth use. Recruitment was facilitated through partnership with the Wakiso District Health Officer (DHO). The research team met with the DHO to describe the study objectives, and the DHO drafted a letter of Introduction to the Health centers for the research team to initiate contact with health centers in the study region. The research team then met with the medical superintendents at health centers in the Central Region of Uganda (Kasangati HCIV, Namulonge Health Centre III, Wattuba Health Centre III, and Buwambo Centre HCIV) to provide the introductory letter from the DHO and to schedule site visits with the designated nurse at each site to determine the best approaches for distributing surveys at each site. Research team members distributed paper copies of the patient and provider surveys at each site visit with the designated nurse. Surveys were distributed to all patients during health care encounters at the health centers during the research team’s site visit. The designated nurse at each site helped distribute and collect the surveys from providers. Through our existing contacts, digital surveys were also emailed to service providers from academia, health IT professionals, and the Infectious Diseases Institute (IDI) from January through June 2022. Given the descriptive nature of this cross-sectional study, we did not conduct power analyses but aimed to have a minimum sample of 50 participants in each group to ensure sufficient data for descriptive analyses.

### Ethical Considerations

This study was reviewed for human subject research ethics and was approved as exempt by the Institutional Review Board at Michigan State University (Study ID 00006715). An informed consent waiver, including a description of the study purpose and contact information for the study investigators, was provided on the study surveys. The consent waiver on the surveys specified that participation was voluntary and that responses were confidential. Surveys were anonymous and did not collect any personally identifiable information. Participants were not compensated for survey completion.

### Data Collection

Study surveys were developed for patients and providers. The patient survey questions were based on the validated Service User Technology Acceptability Questionnaire, which measures user perceptions about the acceptability of telehealth in domains of enhanced care, increased acceptability, privacy and discomfort, care personnel concerns, telehealth as a substitution, and satisfaction [17]. The provider survey was based on a questionnaire developed by the American Medical Association to assess telehealth preferences and impact on clinical practice, including perceptions of clinical value and efficiency of telehealth services [18]. This modified questionnaire was deployed in our previous study assessing telehealth satisfaction among providers in rural Northwest Michigan, United States of America [19]. Informed by the Technology Acceptance Model [20], the surveys also assessed the following factors influencing an individual’s intention to use new technology: perceived usefulness and perceived ease of use. Survey respondents provided perceptions of telehealth using a 5-point Likert scale, where 1 indicated “strongly agree” and 5 indicated “strongly disagree.” Additionally, the survey ascertained demographic information, including age range (<30, 30‐49, 50‐65, and 65+ years), gender, length of time in practice (providers only), and self-reported health status (patients only). The survey also asked whether respondents had heard about telemedicine and used telemedicine prior to completing the survey. The provider survey also ascertained information on barriers to telehealth using the question: “What are your biggest challenges, if any, that you face regarding telehealth visits? Select all that apply.” The following options were available for selection, lack of reimbursement, licensure, technology challenges for the patients, technology challenges for the health care provider/practice, low patient engagement, lack of implementation support, no challenges, or other. This list was based on a prior review of barriers to telehealth in Sub-Saharan Africa [21], with reimbursement issues reflecting barriers due to differential financial return for telehealth visits and licensure indicating legal and regulatory barriers to telehealth implementation. Survey data were entered into Qualtrics, either directly by participants completing the online survey or by study staff for paper-based surveys.

### Statistical Analysis

The survey data were downloaded from Qualtrics and collated in an Excel spreadsheet for analysis. We conducted descriptive statistics of the survey items, describing participant characteristics, perceptions of telehealth, and barriers to using telehealth among providers. Descriptive statistics were calculated to describe provider-reported barriers to telehealth. We also assessed whether telehealth perceptions varied according to participant age, and previous telehealth use, length of time practicing medicine (providers only), and health status (patients only) using Student *t* tests. In secondary analyses, we assessed patient perceptions among the subset of individuals who had used telehealth previously. Missing data were excluded from analysis. All statistical analyses were performed using SAS (version 9.4; SAS Institute) and statistical significance was defined at *P*<.05.

## Results

Surveys from 61 providers and 91 patients were completed and included in the analysis. For providers, 41 surveys were completed on paper and 20 were completed online. For patients, all 91 surveys were completed on paper. As shown in Table 1, most providers were between the ages of 30‐49 years (n=43, 71.1%) and reported female gender (n=38, 63.3%). Providers varied in the length of time in practice, with 20% (n=12) of respondents practicing less than 5 years, 43.3% (n=25) practicing between 5 and 10 years, and 36.7% (n=22) practicing more than 10 years. Most of the surveyed providers had heard about telemedicine before (n=42, 68.9%) and used telemedicine previously (n=48, 78.7%). Of note, several providers (n=6) noted that they had not heard of telemedicine previously but reported using telemedicine after reading the description that telemedicine included telephone-based visits. Patient survey respondents were younger than providers, with 57.8% (n=52) of patients under the age of 30 years. Most patient participants were female (n=67, 74.4%) and reported health status as “Excellent” or “Very good” (n=26, 28.9% and n=23, 25.6%, respectively). Compared to providers, a lower percentage of patients had heard of telemedicine (n=32, 35.2%) or used telemedicine previously (n=18, 19.8%).

**Table 1.** Characteristics of provider and patient survey respondents. Missing data on age, gender, length of time in practice and self-reported health status for 1 patient survey.

	Providers (n=61), n (%)	Patients (n=91), n (%)
Age, years		
<30	10 (16.7)	52 (57.8)
30‐49	43 (71.7)	22 (24.4)
50‐65	6 (10)	10 (11.1)
>65	1 (1.7)	6 (6.7)
Gender		
Female	38 (63.3)	67 (74.4)
Male	22 (36.7)	23 (25.6)
Length of time in practice, years		
<5	12 (20)	—^a^
5‐10	25 (43.3)	—
11‐20	16 (26.7)	—
>20	6 (10.0)	—
Self-reported health status		
Excellent	—	26 (28.9)
Very good	—	23 (25.6)
Good	—	17 (18.9)
Fair	—	20 (22.2)
Poor	—	4 (4.4)
Heard about telemedicine previously		
Yes	42 (68.9)	32 (35.2)
No	19 (31.2)	59 (64.8)
Used telemedicine previously		
Yes	48 (78.7)	18 (19.8)
No	13 (21.3)	73 (80.2)

a Not applicable

**Figure 1.**
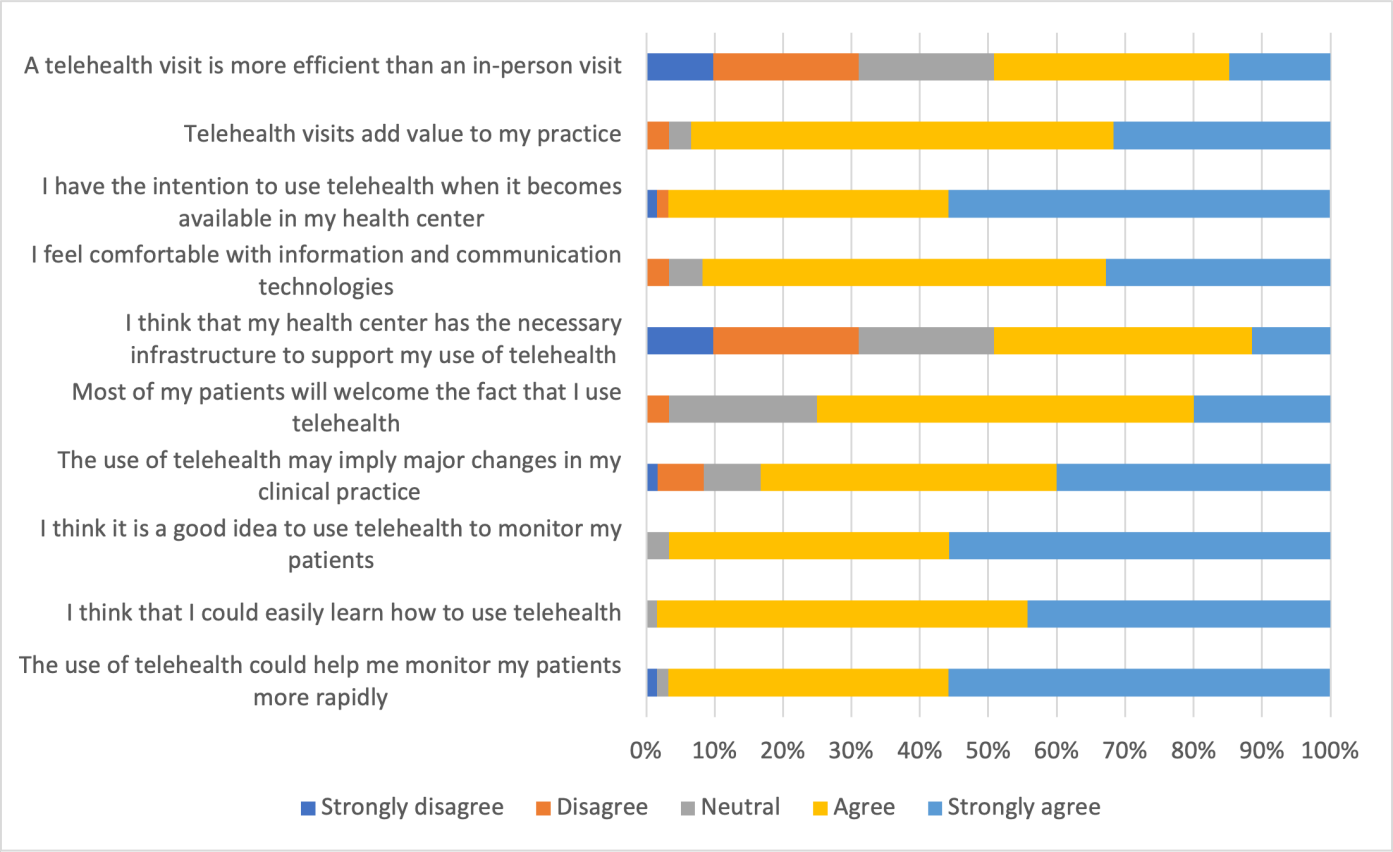
Provider perceptions of telehealth from a cross-sectional survey conducted in Central Uganda, January 2022-July 2022 (n=61).

As shown in Figure 1, provider perceptions of telehealth were generally positive, with 96.7% (n=59) agreeing or strongly agreeing that telehealth could help them monitor patients more rapidly and 98.4% (n=60) reporting that they could easily learn how to use telehealth. While 93.4% (n=57) reported that telehealth adds value to clinical practice, less than half (n=30, 49.2%) felt that telehealth was more efficient than in-person visits. Most providers (n=56, 91.8%) reported feeling comfortable with information and communication technologies, but only 49.2% (n=30) thought their health center had the necessary infrastructure to support telehealth use. Indeed, 83.3% (n=51) of providers reported that telehealth use would imply major changes in their clinical practice. Finally, all 48 providers who had used telehealth would recommend telehealth services to others, and all 13 providers who had not used telehealth would be interested in using telehealth services in the future.

**Figure 2.**
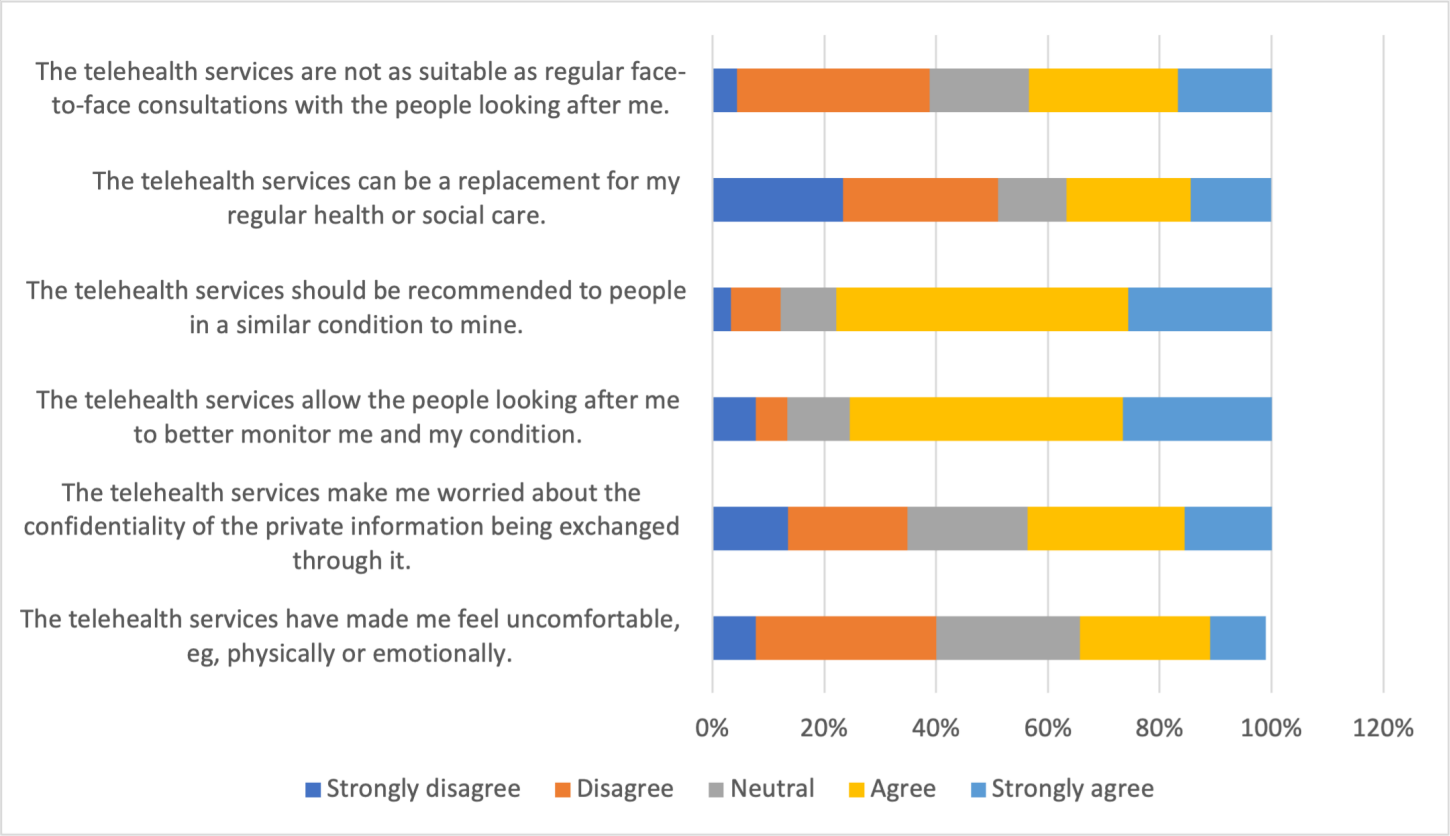
Patient perceptions of telehealth from a cross-sectional survey conducted in Central Uganda, January 2022-July 2022 (n=91).

Perceptions of telehealth were less favorable among patients compared to providers (Figure 2). Nearly 78% (n=70) agreed or strongly agreed that telehealth services should be recommended to people in a similar condition, and 76% (n=68) agreed or strongly agreed that telehealth services would allow their caretakers to better monitor their health condition. Although approximately 33% (n=30) of patient survey respondents agreed or strongly agreed that telehealth services have made them feel uncomfortable, and 43.8% (n=39) agreed or strongly agreed that telehealth services make them worried about the confidentiality of private information. Roughly half of the respondents (n=46, 51.1%) did not agree that telehealth could replace regular care, and 43.4% (n=39) agreed or strongly agreed that telehealth services are not as suitable as regular face-to-face consultations. In secondary analyses among the subset of patients who had used telehealth previously (n=18), favorable perceptions were more pronounced, with nearly 90% (n=82) reporting that telehealth services increased their access to care, 89% (n=81) reporting that telehealth saved time, and 88% (n=80) reporting that telehealth services have helped them improve their health. Over 72% (n=66) of patients who had used telehealth previously reported satisfaction with the telehealth services they received. All 18 patients who had used telehealth would recommend telehealth services to others. However, only 27.8% (n=25) of those who had used telehealth agreed or strongly agreed that telehealth services allowed them to be less concerned about their health status (data not shown). Of the 73 patients who had not used telehealth, 60 (82.2%) would be interested in using telehealth services in the future.

Provider-reported barriers to telehealth, shown in Table 2, included technology challenges for the patient (34/132, 26%), low patient engagement (25/132, 19%), and lack of implementation support (24/132, 18%). Lack of reimbursement (19/132, 14%) and technology challenges for the health care provider or practice (19/132, 14%) were also noted.

**Table 2.** Provider-reported barriers to telehealth from a cross-section survey conducted in Central Uganda, January 2022-July 2022.

	Number of provider-reported barriers (n=132), n (%)
Lack of reimbursement	19 (14)
Licensure	5 (4)
Technology challenges for the patient	34 (26)
Technology challenges for the health care provider or practice	19 (14)
Low patient engagement	25 (19)
Lack of implementation support	24 (18)
No challenges	2 (2)
Other	4 (3)

Overall, provider perceptions of telehealth did not differ according to age, gender, length of time practicing medicine, or based on prior telehealth use (Table 3). However, intentions to use telehealth when it becomes available were slightly higher among male versus female providers (4.73 vs 4.34 on Likert 1-5 scale; *P*=.03). Conversely, several differences in perceptions of telehealth according to patient characteristics were observed (Table 4). For example, concerns about confidentiality with telehealth were slightly higher among patients who did not use telehealth compared to users (3.29 vs 2.35; *P*=.01). Additionally, patients with better self-reported health status were more likely to agree that telehealth should be recommended to people in a similar condition to theirs compared to those with poorer health status (4.14 vs 3.53; *P*=.003). Although not statistically significant, males were slightly more likely than females to report feeling uncomfortable with using telehealth (3.30 vs 2.80; *P*=.06). Interest in participating in telehealth visits in the future was higher among younger (<50 y) versus older participants (3.22 vs 2.56; *P*=.07), among females versus males (4.08 vs 3.43; *P*=.05) and among those with better versus worse self-reported health status (4.12 vs 3.64; *P*=.04).

**Table 3.** Provider perceptions overall and according to provider characteristics.

	Overall (n=61), mean (SD)	Age			Sex			Length of time practicing			Telehealth use		
		<50 years (n=53), mean (SD)	≥50 years (n=7), mean (SD)	*P* value	Male (n=22), mean (SD)	Female (n=38), mean (SD)	*P* value	≤10 years (n=37), mean (SD)	>10 years (n=22), mean (SD)	*P* value	Used telehealth (n=48), mean (SD)	Did not use telehealth (n=13), mean (SD)	*P* value
The use of telehealth could help me monitor my patients more rapidly	4.49 (0.7)	4.47 (0.7)	4.57 (0.5)	.73	4.41 (0.6)	4.53 (0.8)	.54	4.58 (0.6)	4.32 (0.9)	.22	4.52 (0.7)	4.38 (0.5)	.54
I think that I could easily learn how to use telehealth	4.43 (0.5)	4.42 (0.5)	4.57 (0.5)	.47	4.45 (0.5)	4.42 (0.6)	.82	4.45 (0.6)	4.41 (0.5)	.79	4.38 (0.5)	4.62 (0.51)	.15
I think it is a good idea to use telehealth to monitor my patients	4.52 (0.6)	4.49 (0.6)	4.71 (0.5)	.33	4.55 (0.5)	4.50 (0.6)	.77	4.47 (0.6)	4.59 (0.5)	.45	4.48 (0.6)	4.69 (0.5)	.23
The use of telehealth may imply major changes in my clinical practice	4.13 (0.9)	4.15 (0.9)	4.43 (0.5)	.43	4.14 (0.9)	4.22 (0.8)	.73	4.24 (0.8)	4.09 (0.9)	.52	4.09 (1.0)	4.31 (0.9)	.65
Most of my patients will welcome the fact that I use telehealth	3.92 (0.7)	3.94 (0.8)	3.71 (0.8)	.45	3.82 (0.7)	3.97 (0.8)	.45	3.84 (0.8)	4.05 (0.7)	.32	4.00 (0.8)	3.62 (0.5)	.10
I think that my health center has the necessary infrastructure to support my use of telehealth	3.20 (1.2)	3.19 (1.2)	3.14 (1.1)	.93	3.18 (1.2)	3.18 (1.2)	.99	3.13 (1.3)	3.27 (1.1)	.66	3.27 (1.2)	2.92 (1.2)	.36
I feel comfortable with information and communication technologies	4.21 (0.7)	4.21 (0.7)	4.29 (0.8)	.78	4.18 (0.7)	4.24 (0.7)	.77	4.29 (0.7)	4.09 (0.7)	.29	4.23 (0.7)	4.15 (0.6)	0.73
I have the intention to use telehealth when it becomes available in my health center	4.48 (0.7)	4.43 (0.8)	4.86 (0.4)	.16	4.73 (0.5)	4.34 (0.8)	.03	4.53 (0.6)	4.41 (0.9)	.56	4.46 (0.7)	4.54 (0.9)	.73
Telehealth visits add value to my practice	4.22 (0.7)	4.21 (0.7)	4.29 (0.5)	.79	4.18 (0.7)	4.24 (0.6)	.74	4.13 (0.7)	4.38 (0.6)	.17	4.21 (0.7)	4.23 (0.8)	.93
A telehealth visit is more efficient than an in-person visit	3.23 (1.2)	3.28 (1.2)	3.00 (1.4)	.57	2.95 (1.2)	3.42 (1.2)	.16	3.21 (1.2)	3.32 (1.3)	.75	3.23 (1.2)	3.23 (1.4)	.99

**Table 4.** Patient perceptions overall and according to patient characteristics.

	Overall (n=91), mean (SD)	Age			Sex			Health status			Telehealth use		
		<50 years (n=74), mean (SD)	≥50 years (n=16), mean (SD)	*P* value	Male (n=23), mean (SD)	Female (n=67), mean (SD)	*P* value	<Very good (n=41), mean (SD)	≥Very good (n=49), mean (SD)	*P* value	Used telehealth (n=18), mean (SD)	Did not use telehealth (n=73), mean (SD)	*P* value
The telehealth services have made me feel uncomfortable, eg, physically, or emotionally.	2.96 (1.1)	3.74 (1.1)	3.75 (1.0)	.97	3.30 (1.1)	2.80 (1.1)	.06	2.98 (1.0)	2.90 (1.2)	.75	2.56 (1.1)	3.06 (1.1)	.09
The telehealth services make me worried about the confidentiality of the private information being exchanged through it.	3.11 (1.3)	3.94 (0.9)	3.50 (0.9)	.08	3.09 (1.0)	3.11 (1.4)	.96	2.90 (1.4)	3.27 (1.2)	.18	2.35 (1.1)	3.29 (1.3)	.01
The telehealth services allow the people looking after me to better monitor me and my condition.	3.81 (1.1)	3.88 (1.0)	3.56 (0.8)	.24	4.00 (1.1)	3.76 (1.1)	.38	3.70 (1.2)	3.92 (1.1)	.37	4.06 (1.2)	3.75 (1.1)	.31
The telehealth services should be recommended to people in a similar condition to mine.	3.88 (1.0)	2.86 (1.1)	3.25 (0.9)	.21	3.65 (1.1)	3.94 (1.0)	.24	3.53 (1.1)	4.14 (0.9)	.003	4.11 (0.7)	3.82 (1.1)	.16
The telehealth services can be a replacement for my regular health or social care.	2.77 ( 1.4)	3.14 (1.3)	3.38 (0.9)	.48	2.96 (1.4)	2.68 (1.4)	.42	2.75 (1.4)	2.76 (1.4)	.99	3.11 (1.6)	2.68 (1.4)	.25
The telehealth services are not as suitable as regular face to face consultations with the people looking after me.	3.17 (1.2)	3.88 (0.9)	3.50 (0.9)	.15	3.26 (1.3)	3.14 (1.2)	.67	3.28 (1.1)	3.08 (1.3)	.46	2.89 (1.3)	3.24 (1.2)	.28
I would be interested in continuing to participate in telehealth visits in the future.	3.92 (1.1)	3.22 (1.3)	2.56 (1.3)	.07	3.43 (1.1)	4.08 (0.9)	.05	3.64 (1.1)	4.12 (1.0)	.04	4.11 (1.0)	3.87 (1.1)	.42

## Discussion

Findings from this study demonstrate generally favorable perceptions of telehealth among providers and patients in Central Uganda. Providers were more likely than patients to have used telehealth, and most noted the clinical value of telehealth and reported feeling comfortable using the technology. Importantly, this study also identified provider barriers impeding telehealth use, including technology challenges for the patient and the lack of infrastructure and implementation support. Patient perceptions of telehealth were largely neutral to positive, and concerns around confidentiality of information and comfort level using telehealth services were reported. Overall, our findings suggest the strong potential of telehealth approaches to improve health care service delivery in Central Uganda. Our results demonstrate the need for future telehealth implementation efforts that address technology and infrastructure-related challenges.

Overall, the favorable perceptions of telehealth among providers in this study align with results from another cross-sectional study in Uganda, where attitudes toward telemedicine were overwhelmingly positive, yet gaps in technology readiness were pronounced [22]. Results from the providers in this study demonstrate evidence for perceived usefulness of telehealth in monitoring patient care and providing clinical value to patients. These findings align with results from prior studies [23-25], including our prior study of rural providers in the United States [19]. Additionally, most providers in this study reported ease of use in telehealth technology platforms. However, concerns around the efficiency of telehealth visits compared to in-person clinic visits were also commonly reported. Concerns around the efficiency of telehealth approaches were also noted in our team’s prior study of rural providers in the United States [19]. These findings differ, however, from results in other study settings where improvements in efficiency with telehealth approaches were observed [23,24]. The lack of supportive infrastructure for telehealth visits, which was noted as an important barrier to telehealth among survey respondents, may contribute to the discrepancies in findings across studies. Indeed, most providers in this study reported that telehealth approaches would require major changes in current clinical practice and workflows. Such changes may help improve overall efficiencies with the telehealth system and overcome this reported barrier.

In this study, patient perceptions of telehealth were less favorable compared to providers. This may be due in part to lower levels of telehealth utilization and comfort in using the technology among patients. Indeed, while most providers in this study had used telehealth, both awareness and telehealth use among patients were much lower. This suggests the need to better understand patient-level barriers to telehealth use and to target patient education efforts to increase overall knowledge about telehealth applications from the patient perspective. Importantly, our findings also demonstrate satisfaction among patients who had previously used telehealth, and most previous users also recognized the clinical utility of telehealth for care monitoring. However, there were concerns among patients related to the comfort of using telehealth and the confidentiality of the private information shared through the technology. These findings emphasize the need for future efforts to increase awareness of telehealth services, offer educational trainings on digital literacy, and address concerns related to the confidentiality of information.

Perceptions of telehealth in this study did not differ substantially by most provider and patient characteristics. Interestingly, we did not observe any significant differences in telehealth perceptions according to provider or patient age. This differs from findings in U.S. study populations, where telehealth perceptions were more favorable among younger patients and providers [19,26,27]. In these prior studies, age-related differences in telehealth perceptions were largely attributed to lower technology access and digital literacy among older adults [28]. Participants in this study were younger overall, and this may have contributed to the lack of observed differences in telehealth perceptions by age in our study. Intentions to use telehealth in clinical practice were modestly higher among male compared to female providers in our study. This finding differs from those assessing mental health providers in the United States, where no gender differences in intention to continue using telehealth after the COVID-19 pandemic were observed [29]. It is important to note that the intention to use telehealth was high among female and male providers in our study. Thus, future studies are needed to assess whether this gender difference persists in other study settings. Interestingly, patients with better self-reported health status were more favorable about recommending telehealth to people in similar conditions and were also more interested in continuing to participate in telehealth visits in the future. These findings highlight the growing recognition that telehealth may not be appropriate or feasible for all patients or all clinical care situations [30].

The strengths of this study include the focus on multiple perspectives, including those of both providers and patients across multiple clinical sites in this understudied region. Moreover, our study used a community-engaged approach, working in partnership with community members and incorporating input on study processes and outcomes from clinical and community partners. This approach can help guide implementation efforts toward those with the most community relevance and potential impact. We also held dissemination workshops in the community to discuss the findings and inform next steps to ensure that research efforts address the relevant needs and preferences of the community members in this region of Uganda. This research provides a replicable and scalable model that can help inform telehealth implementation efforts in other under-resourced settings. This study did have several limitations. First, using a convenience sampling strategy, we were unable to calculate a response rate given resource limitations for tracking survey distribution across multiple study sites. While the survey development was informed by the technology acceptance model [20] and modeled on surveys used in prior studies [31,32], the survey questions were not specifically validated. Additionally, we were unable to assess whether perceptions of telehealth differed according to the type of health care provider, as this information was not collected on the provider survey. This should be assessed in future studies, particularly given that readiness to integrate telemedicine services varied across health facility types and job roles in a prior study conducted in Uganda [14]. Finally, this study focuses on a single region in Uganda, and results may not be broadly generalizable across other populations and settings.

Overall, our findings demonstrate both satisfaction with telehealth among users and willingness to use it among nonusers for both providers and patients. Indeed, providers and patients who previously used telehealth unanimously reported recommending the service to others. All surveyed providers and most patients who had not used telehealth were interested in using this approach in the future. These findings suggest positive attitudes and behavioral intentions to use telehealth, which are key components to technology acceptance according to the technology acceptance model [20]. Taken together, our results suggest that the perceived usefulness, ease of use, and attitudes toward telehealth are supportive of future implementation efforts to increase the reach of telehealth services in Uganda and in other low- and middle-income countries.

Findings from this study can inform the implementation of acceptable telehealth approaches to address disparities propagated by health care access barriers in Sub-Saharan Africa.

## Abbreviations

DHO: District Health Officer
